# Characteristics of online user-generated text predict the emotional intelligence of individuals

**DOI:** 10.1038/s41598-023-33907-4

**Published:** 2023-04-25

**Authors:** Yaniv Dover, Yair Amichai-Hamburger

**Affiliations:** 1 The Hebrew University Business School, Jerusalem, Israel; 2 The Federmann Center for the Study of Rationality, Jerusalem, Israel; 3 The Research Center for Internet Psychology, Reichman University, Herzliya, Israel

**Keywords:** Psychology, Human behaviour, Computational science

## Abstract

Emotional intelligence is a well-established indicator of performance and the ability to maintain successful social relationships. Moreover, it is potentially an important factor in social dynamics occurring on large digital platforms, e.g., opinion polarization, social conflict, and social influence. Users publicly exchange enormous amounts of text on digital platforms, which can potentially be used to extract real-life insights. Yet, currently, the prevalent approach to measuring emotional intelligence uses mainly self-report surveys and tasks—considerably limiting the feasibility of real-life large-scale studies. We analyze the online public texts of users, who also completed emotional intelligence measures, to find that characteristics of online public texts can be used to predict emotional intelligence at a level like that of commonly used psychometric indicators (e.g., SATs) to predict real-life outcomes. For example, we find that high emotional intelligence individuals consistently use more positive-affect language, less negative-affect language and use more social-oriented language than low emotional intelligence individuals. Our findings provide insight into the role of personality on digital platforms and open the possibility of studying emotional intelligence in large and diverse real-life data. To support the use of online public text as a tool to research emotional intelligence, we provide an anonymized version of the data.

## Introduction

Emotional intelligence (EI) is one of the leading indicators of the ability to recognize and understand one's own emotions and the emotions of others, which is related to the ability to manage one's own behavior and relationships effectively. Higher emotional intelligence has been shown to predict more positive social relations in both childhood^1,2^ and adulthood^3^, better academic outcomes^4,5^, and stronger performance in the workplace^6^. In terms of research, EI is a useful construct which helps explain how emotions and emotion regulation affects decision-making processes and individual-level and social behavior. The importance of EI in practical applications is also extensively demonstrated as a useful tool that helps individuals and organizations better understand and manage emotions in organizational contexts, which can lead to improved communication, teamwork, and overall effectiveness in both personal and professional relationships. While other similar indicators exist, such as empathy^7,8^, self-regulation^9,10^, and social skills^11,12^, EI remains an intensive topic of research^5,13–17^ and a widely used instrument across organizations^18^. Given that EI is an important skill and indicator in the real world, the growing availability of public online texts can be an opportunity for both research and practice purposes. Online communication has become an integral part of our daily lives, and we increasingly rely on it for interactions and socializing with others. Social media platforms, discussion forums, and messaging apps allow us to connect with people from all over the world, and to share our thoughts, experiences, and ideas in real-time. Online communication also plays a significant role in day-to-day economic interactions. Social media, e-commerce platforms, and gig economy websites have transformed the way we do business and work. Hence, understanding the impact of online communication on both social and economic interactions is crucial for developing effective policies and strategies to support the growth and development of digital technologies. Therefore, as online communication becomes more prevalent, it is crucial for researchers and practitioners to understand the role of emotional intelligence in online communication patterns. Emotional intelligence has been shown to be strongly linked to effective communication, which is also critical in online contexts, and therefore there is a paramount need for understanding its role in online communication, and how it is reflected in online texts.

Real-life user-generated text on online platforms is potentially a rich measure of behavior, and is likely reflective of the author’s characteristics, but it is unclear whether this can be used specifically as a measure of EI. The purpose of this work is to test whether an individual’s public online text can be predictive of their EI traits. A similar approach was used to demonstrate that online public texts can be used to estimate the five factor model of personality traits^19,20^. Despite the proven importance of EI as a behavioral indicator, there is a scarcity of work that attempts to detect it from online public texts. Here, we address this gap in the literature.

Most of the extant methods that measure EI are survey based, i.e., they require individuals to respond by filling out a survey or by performing specialized tasks. Therefore, these methods are difficult to use in real-life contexts, and with large data and large groups of people. Therefore, if indeed it is possible to extract the EI of online users from their online text, there are several potential implications for both research and practice. First, if public online texts do predict EI, this can allow studying EI in a large and diverse dataset which can provide a more comprehensive understanding of how EI influences behavior and communication in a variety of digital platform contexts. It can also enable the identification of trends or patterns in EI over time, which can inform the development of interventions or strategies to improve dynamics in online platforms. Digital platforms are increasingly becoming a major outlet through which people interact with others for informational, recreational, work-related, and transactional purposes. The social and emotional dynamics in digital platforms have been shown to affect the decision-making process of users, such as in decisions about health^21,22^, voting^23^, and consumption^24^. The effects of social media on conflict and polarization is another topic that is becoming a focus in the literature^25^ and in popular media^26^. Therefore, understanding the role of the EI of individuals within the social dynamics on digital platforms, in real time, has the potential to inform the design, policy planning, and moderation of these platforms to optimize social interactions within them. Insight into this topic could have practical outcomes and help increase the welfare of all involved parties, including the platform users, themselves. Organizations may use text analysis to assess the EI of candidates for employment or to identify areas for improvement in their employees' EI. In sum, it is important to study whether user-generated online text can be used to estimate the EI of its author. To test whether online public texts can predict EI, we asked 681 users of the popular discussion platform *Reddit* to complete the WLEIS^27^ emotional-intelligence questionnaire, which measures four aspects of EI: self-emotion appraisal, others’ emotion appraisal, use of emotion, and emotion regulation. We also asked these users to volunteer their online public texts on the platform. This allowed us to extract the characteristics of their texts and compare it to their EI, thus, testing whether there is any relationship between the two.

## Literature review

### Emotional intelligence: an overview

One of the earliest attempts to define the ability of individuals to manage their emotions was done by Salovey and Mayer^28^, who described EI as “the subset of social intelligence that involves the ability to monitor one's own and others' feelings and emotions, to discriminate among them, and to use this information to guide one's thinking and actions.” (p. 189). Mayer et al. followed the conceptualization developed by Salovey and Mayer^29^ and defined EI as a set of interrelated skills that can be classified within the following four dimensions: the ability to perceive accurately, appraise, and express emotion; the ability to access and/or generate feelings when they facilitate thought; the ability to understand emotion and emotional knowledge; and the ability to regulate emotions to promote emotional and intellectual growth. Davies et al.^30^ used the extant literature to also develop four dimensions of emotional intelligence, and 2 years later, Mayer et al.^31^ developed the Multifactor Emotional Intelligence Scale. Although the definitions of EI used by Davies et al. and Mayer et al.^31^ are not identical, the differences in the definitions are minor. In this study, we use the four-dimensional definition of EI developed by Davies et al.: (1) Appraisal and expression of emotion in oneself—this relates to an individual's ability to understand his or her deep emotions and to be able to express emotions naturally. People who have good ability in this area will sense and acknowledge their emotions better than most others. (2) Appraisal and recognition of emotion in others—this relates to an individual's ability to perceive and understand the emotions of the people around them. People who rate highly in this ability will be very sensitive to the emotions of others, as well as be able to predict others' emotional responses. (3) Regulation of emotion in oneself—this relates to the ability of a person to regulate his or her emotions, enabling a more rapid recovery from psychological distress. A person with high ability in this area is able to quickly return to a normal psychological state after rejoicing or becoming upset. Such a person would also have better control of his or her emotions and would be less likely to lose his or her temper. (4) Use of emotion to facilitate performance—this relates to the ability of a person to make use of his or her emotions by directing them toward constructive activities and personal performance. A person who is highly capable in this dimension is able to encourage him- or herself to do better continuously. They would also be able to direct their emotions in positive and productive directions. We use Davies et al.'s definition of EI because it is more representative of the EI literature. While Davies et al.'s review considered Mayer and Salovey's definition of EI, and is in fact, quite similar, it also matches well with the summary of Ciarrochi et al.^32^ of the four basic areas of EI. Davies et al.'s definition of the dimensions of EI allows us to focus on the nature and characteristics of the EI construct.

For our purposes, here, we adopt a widely-accepted and validated scale (WLEIS^27^) that allowed us to survey the four dimensions described above: (1) the Self-Emotion Appraisal (SEA)—the capacity of the individual to understand their own emotional behavior, (2) the Others' Emotion Appraisal (OEA)—the capacity to understand the use of emotions by other people, (3) the Use of Emotion (UOE)—the extent to which an individual uses emotion in their daily life, and (4) the Regulation of Emotion (ROE)—the extent to which an individual can regulate their own emotions.

### Public online texts as a tool to study emotional intelligence in digital platforms

Online text communication has become an essential part of modern life. It allows people to connect and communicate with others, conduct business, access information, express themselves publicly, and engage in entertainment. Whether it is through social media, email, messaging apps, or online forums, online text communication has revolutionized the way we interact with each other and the world around us. As such, it has become central to modern life, facilitating social connections, knowledge sharing, and economic growth. Therefore, online texts offer the opportunity to be a rich and credible source for research into human behavior. Thus, real-life user-generated text is potentially a rich measure of behavior, and is probably reflective of user characteristics. Therefore, our main question here is whether it can be used as a measure of emotional intelligence. The possibility of using an individual’s text to assess their EI is appealing both in terms of research and practice. It opens up the opportunity to study EI in large and diverse data, in real-life settings, and with high temporal resolution. A similar approach was used to demonstrate that text can be used to measure the “Big Five” personality traits^19,20^. To predict these traits from public online texts, digital platform users were asked to fill out questionnaires. In parallel, researchers analyzed the texts that users produced on digital platforms and then tested whether the characteristics of these texts were correlated with the Big Five traits. Correlations were indeed found. Yet, despite the importance of EI as a behavioral indicator, there is a scarcity of work that attempts to detect it from user-generated publicly available online texts. Here, we address this gap in the literature.

### How can text be characterized?

In order to extract text characteristics, there are several options for researchers. One of the most popular leading tools is the well-known Linguistic Inquiry and Word Count (LIWC) software^33^ with built-in theme dictionaries to analyze users’ texts. LIWC uses pre-validated dictionaries of words in specific topics to count how many words appear in a certain text. For example, one of the LIWC dictionaries can be used to count words of positive emotion and another dictionary to count words of negative emotion. The LIWC dictionaries have been validated^34^, and with almost 6000 citing works, it is one of the more extensively used text analysis tools in applied psychology research. For example, previous work used LIWC to demonstrate that text characteristics correlate with the five factor model personality traits^20^, mental health^35^, elections using social media^36^, corporate culture^37^, and countless other phenomena. We used the LIWC 2022 version^38^, which can extract variables automatically from large numbers of texts. The variables that LIWC produces are divided into four main categories: (1) summary variables, (2) linguistic dimensions, (3) psychological processes, and, (4) expanded dictionaries. The expanded dictionaries include variables pertaining to culture, lifestyle, physical aspects, personal states, motives, perception, time focus, and conversational variables. A list of the relevant variables to our study is given in Appendix S4. Given that it is possible to decompose public online texts into measurable characteristics, and given that prior work found that text can correlate with personality, our hypothesis is:

H1: Characteristics of text produced in online platforms can exhibit correlation with dimensions of emotional intelligence.

If text produced on online platforms is an expression of people’s thoughts, behaviors, and needs—it is interesting to hypothesize which aspects of it could predict EI. The literature demonstrates that EI is a measure of the ability of an individual to manage their emotions, perceive other people’s emotions, and be capable of sustainable social interactions. Therefore, it is plausible to a-priori expect that aspects related to social and emotional process in texts would be predictive of EI:

H2: Characteristics of text produced on online platforms related to affect and social processes should exhibit correlation with dimensions of emotional intelligence.

An important question regarding online texts and EI is what we expect the correlation patterns to be for positive or negative affect. Our baseline expectation is that positive affect in text will be positively correlated with EI because high EI individuals are known to appreciate the value of positive communications. Yet, it is a-priori unclear how negative affect in texts will correlate with EI. If people use online texts mainly for productive communication, then the basic expectations is that high EI individuals will use less negative affect, and for the same reasons, they are expected to use more positive affect in communications. But it may be the case that individuals use online texts mainly to regulate emotions, e.g., to vent. If this is the dominant use, then we would expect that high EI will correlate with an increased use of negative affect in texts:

H3: Characteristics of text produced on online platforms related to positive affect should exhibit positive correlation with dimensions of emotional intelligence, while characteristics of text related to negative affect should exhibit negative correlation with dimensions of emotional intelligence.

Although we expect that the affect and social processes characteristic of the text will correlate with EI, it is less clear whether other text characteristics could be predictive of EI. Yet, EI was shown to be, directly or indirectly, related to a wide range of day-to-day aspects of life. Therefore, there is reason to suspect that other aspects of text can be predictive of EI. For example, if a person produces text on online platforms that expresses an increased interest in culture (i.e., measured through the Culture dictionary of LIWC), it may be a sign that this person is interested in social issues, perhaps suggesting their EI is high. Texts that are higher on issues pertaining money (another LIWC dictionary) may suggest a lower EI. In summary, it is of interest to see whether text characteristics that do not directly measure social- or affect-related aspects, predict EI. To test this, we exploit the structure of LIWC’s text categories, which are arranged under several meta-categories^38^. In Table S5 (supplementary materials) we denote the meta categories that are not directly related to social and affect. The meta-categories are: text summary variables, Linguistic dimensions, Culture, Lifestyle, Physical, Time orientation, Human states, Human motives, Perception and Psychological processes (that are not directly related to social and affect). For example, LIWC categories ‘reward’, ‘risk’ and ‘allure’ fall under the “motives” meta category, while “work” and “money” fall under the Lifestyle category (Table S5). We test whether variables under the above meta-categories which describe the day-to-day lives of people—predict EI.

H4: Characteristics of text produced on online platforms related to meta-categories: Culture, Lifestyle, Physical, Time orientation, Human states, Motives, Text summary variables, Linguistic dimensions, Perception and Psychological processes (that are not directly related to social and affect processes)—should exhibit a correlation with dimensions of EI.

## Data and methods

### Participants

We recruited 681 users from the online platform Reddit. Participants were not sampled randomly, but were Reddit users who voluntarily responded to our posts in several online communities (subreddits) which asked for respondents. Only 618 participants reported gender, out of which 47% indicated they were female, 45% male, and 8% indicated “other.” A total of 613 participants reported their age: 38% indicated they were in the range of 18–24 years old, 40% indicated they were 25–34 years old, 13% indicated 35–44 years old, and 9% indicated they were 45 and older. About 60% of the sample reported that they resided in the United States, 7% reported residence in the United Kingdom, 7% in Canada, and the rest of the 26% reported residence in other parts of the world (including Asia, Africa, South America Europe, and the Middle East). Out of the 681 participants, only 606 completed both their EI measures and volunteered their online public texts.

### Measures

#### Emotional intelligence

The measure we used to assess emotional intelligence of the participants was the Wong and Law Emotional Intelligence Scale (WLEIS; Wong & Law, 2002): The scale consists of 16 items (see Appendix S3), and the answers were obtained according to a seven-point Likert scale, ranging from 1 (strongly disagree) to 7 (strongly agree) to measure the users’ perception of their EI, distributed among four dimensions: Self-Emotional Appraisal (SEA), 4 items (α = 0.86; e.g., I have a good sense of why I feel certain feelings most of the time); Others’ Emotional Appraisal (OEA), 4 items (α = 0.88; e.g., I always know my friends’ emotions from their behavior); Use of Emotion (UOE), 4 items (α = 0.81; e.g., I am a self-motivated person); and Regulation of Emotion (ROE), 4 items (α = 0.81; e.g., I have good control of my emotions). The internal consistency for all scales in this study was α = 0.87. Table 1 provides some summary statistics for the relevant four dimensions in our sample.

**Table 1:** Descriptive statistics of the collected sample of subjects from Reddit. Descriptive statistics of the Reddit sample.

	# of observations	Average (SD)	Minimum	Maximum
Self-appraisal of emotion	606	5.03 (1.21)	1	7
Others’ appraisal of emotion	606	5.06 (1.23)	1	7
Use of emotion	606	4.53 (1.29)	1	7
Regulation of emotion	606	4.87 (1.17)	1	7

#### Participants’ public online texts—Reddit

The user-generated text data we used was collected through the Reddit digital platform^39^. Reddit is a highly popular platform which hosts online discussions and content sharing based around thematic communities, with reportedly more than 52 M users. In Reddit, users write posts combined with text, links, and images that other users can comment on. Essentially, a major purpose of Reddit is to be a platform that hosts interactions between users via user-generated content. This fits our purpose of using text that users use much of the time for social interaction on the platform, as it has the potential to reflect on their EI traits.

#### Extracting text characteristics

As described above, we used the 2022 version of the LIWC text analysis software^38^ to extract characteristics from the text that users posted on Reddit. We collected the public text that each of the 606 users volunteered from their profile page on Reddit, and who also reported the WLEIS measures. The text collected for each user included up to 2000 of their recent posts.We then collated all posts of each of the users such that each user was associated with one large body of text that was an amalgamation of all their posts. The mean number of words per user was 30,258 words, and the median was 8375 words. The texts were then processed by LIWC to produce scores for each of the LIWC dictionaries, i.e., the percentage of words of a certain dictionary which appeared in the user’s text. For example, a score of 5 for the Anxiety dictionary (emo_anx) meant that 5% of the words in the text existed in the anxiety LIWC dictionary. Out of all the LIWC dictionaries, 104 variables were relevant to our study and are listed in Appendix S2. Table 2 is a reproduction of the original work^38^ and lists 22 LIWC variables that are listed under the *Affect* and *Social Processes* categories. Our purpose was to use these variables to test H1, H2, and H3. The table lists the technical name of the variable (middle column), its descriptive name, and a few examples of words that have been included in each variable’s dictionary for clarification. For example, the variable emo_neg is a measure of the percentage of words associated with negative emotion, e.g., *bad*, *hate*, and *hurt*. On the other hand, emo_pos measures positive emotion using words like *good*, *love*, and *happy*.

**Table 2:** LIWC variable names, descriptions, and examples of words from each variable’s dictionary for 23 variables that measure social-related and affect-related aspects of texts. Text analysis variables.

Variable name	LIWC variable	Description/sample dictionary words
Affect	Affect	Good, well, new, love
Positive tone	ton_pos	Good, well, new, love
Negative tone	ton_neg	Bad, wrong, too much, hate
Emotion	emotion	Good, love, happy, hope
Positive emotion	emo_pos	Good, love, happy, hope
Negative emotion	emo_neg	Bad, hate, hurt, tired
Anxiety	emo_anx	Worry, fear, afraid, nervous
Anger	emo_anger	Hate, mad, angry, frustr*
Sadness	emo_sad	:(, Sad, disappoint*, cry
Swear words	Swear	Shit, fuckin*, fuck, damn
Social processes	Social	You, we, he, she
Social behavior	Socbehav	Said, love, say, care
Prosocial behavior	Prosocial	Care, help, thank, please
Politeness	Polite	Thank, please, thanks, good morning
Interpersonal conflict	Conflict	Fight, kill, killed, attack
Moralization	Moral	Wrong, honor*, deserv*, judge
Communication	Comm	Said, say, tell, thank*
Social referents	Socref	You, we, he, she
Family	Family	Parent*, mother*, father*, baby
Friends	Friend	Friend*, boyfriend*, girlfriend*, dude
Female references	Female	She, her, girl, woman
Male references	Male	He, his, him, man

### Procedures

Participants were recruited on a voluntary basis by posting requests on Reddit communities. Asking for participation and linking to an online survey. In return, the respondents were promised entry for a raffle for four $50 gift cards, which were awarded after the data collection was completed. In the online survey (using Qualtrics), participants reported their WLEIS measures and agreed to have their public online texts on Reddit collected and analyzed, on the condition that the data would be kept secured and anonymized. The participants were informed that any identifying details in the data would be removed such that no specific user could be identified, and that the general purpose of the study was to ascertain the relationship between text and EI. The study was approved by the ethics committee of the authors’ institution, and the only inclusion criterion was that the participants should be 18 years old or above.

### Ethical considerations

Informed consent was obtained from all subjects and/or their legal guardian(s). All questionnaires were implemented in an anonymous format. This study was approved by the research ethics committee of the Reichman University (August 2nd, 2021). All methods were performed in accordance with the relevant guidelines and regulations.

## Results

### Do social- and affect-related text characteristics predict emotional intelligence?

To test whether social-related and affect-related text characteristics correlate with EI, we calculated the correlation between the 22 social and affect text variables and the WLEIS four dimensions of EI. The results are presented in Table 3.

**Table 3:** Correlations of text variables for each of the four dimensions of EI. Only variables which exhibit correlations that are significant to the 5% percent level (p-values ≤ 0.05) are listed. Correlations between social- and affect-related text variables and emotional intelligence.

LIWC variable	Self-emotion appraisal (SEA)	Others’ emotion appraisal (OEA)	Use of emotion (UOE)	Regulation of emotion (ROE)
Affect	0.07*	0.14***	− 0.02	0.02
Positive tone	0.08**	0.17***	0.08**	0.07*
Negative tone	− 0.03	− 0.05	− 0.20***	− 0.08**
Emotion	0.04	0.17***	0.01	− 0.01
Positive Emotion	0.08*	0.18***	0.10**	0.05
Negative Emotion	− 0.06	0.03	− 0.17***	− 0.11***
Anxiety	− 0.02	0.07*	0.02	− 0.02
Anger	0.02	− 0.02	− 0.13***	− 0.06
Sadness	− 0.09**	0.02	− 0.18***	− 0.16***
Swear words	0.03	0.01	− 0.14***	− 0.07*
Social processes	0.08**	0.15***	0.04	0.04
Social behavior	0.08**	0.15***	0.05	0.05
Prosocial behavior	0.06	0.18***	0.08**	0.06
Politeness	0.06	0.15***	0.07*	0.08*
Interpersonal conflict	0.04	− 0.09	− 0.08**	0.01
Moralization	0.02	− 0.04	− 0.10***	0.02
Communication	0.07*	0.14***	0.05	0.06
Social referents	0.06	0.12***	0.01	0.02
Family	− 0.01	0.02	0.01	− 0.06
Friends	0.04	0.06	0.05	− 0.01
Female References	0	0.13***	− 0.06	− 0.06
Male references	0.03	0.06	− 0.03	0

*p ≤ 0.10, **p ≤ 0.05, ***p ≤ 0.01.

As is clear from the above table, each of the four dimensions of EI exhibits statistically significant correlations with some of the social and affect text variables. For example, the incidence of positive emotion in the text (emo_pos) is positively correlated with each of the dimensions of EI, the highest being a correlation of 0.17 with others’ emotion appraisal (OEA). The incidence of politeness in the text (polite) is correlated with three of the EI dimensions, the highest being 0.15 with OEA. In general, the statistically significant correlations range between − 0.20 and 0.18. For SEA, 7 out of 22 variables show statistically significant correlation, although all of them seem to be relatively weak, i.e., they have magnitudes below 0.10. For OEA and UOE, 11 variables are significant, most of which are above a magnitude of 0.10; and for ROE, 6 variables out of the 22, out of which only two (negative emotion and sadness) have magnitudes higher than 0.10. This first suggests that there is support for H1, i.e., that text characteristics can indeed be predictive of an individual’s EI. But that the correlations with individual text variables can sometimes be relatively weak. Beyond that, the results support H2, i.e., that as expected, some of the social and affect text characteristics correlate with EI.

#### How negative vs. positive affect predicts emotional intelligence

Even though Table 3 demonstrates that not all affect-related variables correlate with EI, there is a pattern for variables that do exhibit correlations. In the table, all negative correlations that are statistically significant are correlations between a negative type of affect in the text and one of the EI dimensions. For example, both negative tone and negative emotion negatively correlate with UOE and ROE, even though the magnitude of the correlation with negative emotion is low at around 0.10. Similarly, anger, sadness, and swear words all exhibit negative correlations above 0.10 with UOE, and some weaker correlations with the rest of the EI dimensions. Consistently, positive emotion and positive tone words correlate positively with OEA, and more weakly with the rest of the EI dimensions. Therefore, we can say that H3 has support from the findings. High EI is associated with a lower use of negative affect in online texts, and higher use of positive affect. One outlier to this is anxiety that shows a weak positive correlation (0.07) between anxiety and OEA, i.e., people who use words related to anxiety more, have a weak tendency to be more aware of others’ emotions.

#### Negative vs. positive social aspects used in text and emotional intelligence

It is interesting to note that, like affect, higher use of positive social words in text (as measured by: prosocial behavior), words of politeness, and words describing or referencing general social behavior (social processes, social behavior) show positive correlations, especially with OEA but also exhibit weak correlations with other EI dimensions. On the contrary, more negative social-oriented words, such as words used in interpersonal conflict or moralization words, exhibit negative correlations, albeit weak, with one of the EI dimensions—UOE. It is worth pointing out here that even though the correlations between separate individual characteristics and EI dimensions can be low, the overall prediction power, when using several characteristics, can be higher and largely equivalent to other known and commonly-used behavioral indicators, as is shown and explained below.

#### How other text characteristics predict emotional intelligence

We expect that the EI of an individual is not only related to social and affect aspects of their text, but also indirectly, to other aspects. In Table 4, we list all text characteristics, out of the total 104 variables we tested, that are correlated with the dimensions of EI at least at a level of significance of p ≤ 0.05. As in Table 3, each variable in Table 4 represents a LIWC category. For example, the variable “fatigue” represents a dictionary of words that are related to fatigue, i.e., the mental and physical state of exhaustion; the variable “technology” represents words related to technology; and the variable “ethnicity” represents words related to different ethnicities. The descriptions of each variable shown in Table 4, along with examples for each category in the table are given in Appendix S5. The table shows that each of the EI dimensions correlates with some of text characteristics, specifically, SAE has 9 correlates; OAE, 17 correlates; UOE, 20 correlates; and ROE, 22 correlates. For example, language of leadership and status (Clout) is positively correlated with all four dimensions of EI. Text related to ethnicity, sexuality, and death is negatively correlated with ROE, while the frequency of words with more than six letters (BigWords) correlates negatively with it. The table shows that most of the correlationa with the individual text characteristics are relatively weak at around 0.10, and only a few of the correlation magnitudes exceed 0.15. Developing hypotheses regarding each of the variables in Table 4 is beyond the scope of this paper, but we can certainly claim support for H4, and a demonstration that online public texts can be an informative and useful tool to study EI in real life and large data.

**Table 4:** Correlations between text variables that are not under the social and affect category and each of the four dimensions of EI. Only variables which exhibit correlations that are significant to the 5% percent level (p-values ≤ 0.05) are listed. Correlations between general category text variables and emotional intelligence.

Self-appraisal of emotion (SAE)		Others’ appraisal of emotion (OAE)		Use of emotion (UOE)		Regulation of emotion (ROE)	
Variable	Correlation	Variable	Correlation	Variable	Correlation	Variable	Correlation
Fatigue	− 0.08**	1st person plural	0.08**	Clout	0.10***	Space	0.17***
Curiosity	0.09**	Negation	− 0.09**	Tone	0.14***	Work	0.16***
Differentiation	− 0.09**	Causation	− 0.09**	3rd person plural	− 0.08**	Prepositions	0.14***
Mental health	− 0.09**	power	− 0.09**	Numbers	0.08**	Lifestyle	0.14***
Lifestyle	0.09**	Clout	0.11***	Negation	− 0.12***	Achievement	0.13***
Achievement	0.10**	1st person singular	0.11***	Discrepancy	0.10***	Perception	0.13***
Clout	0.10**	2nd person	0.10***	Memory	− 0.10***	Big words	0.11***
Memory	− 0.10**	3rd person singular	0.10***	Culture	0.09**	Articles	0.11***
Work	− 0.11***	Affiliation	0.11***	Ethnicity	− 0.08**	Curiosity	0.11***
**p < 0.05, ***p < 0.01		Want	0.10***	Technology	0.1***	Tone	0.10**
		Future focus	0.11***	Lifestyle	0.16***	Clout	0.09**
		Tone	0.15***	Work	0.15***	Determiners	0.09**
		All pronouns	0.12***	Money	0.16***	Drives	0.09**
		Personal pronouns	0.15***	Physical	− 0.08**	Power	0.09**
		Articles	− 0.12***	Sexual	− 0.12***	Attention	0.09**
		Differentiation	− 0.12***	Death	− 0.15***	Physical	− 0.08**
		Politics	− 0.14***	Fatigue	− 0.09**	Death	− 0.09**
		**p < 0.05, ***p < 0.01		Reward	0.09**	Memory	− 0.10**
				Curiosity	0.08**	Ethnicity	− 0.10**
				Attention	0.1***	Sexual	− 0.10**
				**p < 0.05, ***p < 0.01		1st person singular	− 0.11***
						Mental health	− 0.13***
						**p < 0.05, ***p < 0.01	

#### How much of the variance in emotional intelligence do text variables explain?

An important aspect of the correlation between text variables and EI is the percent of the EI variation that the text variables explain. The more variation that is explained, the higher the text “prediction power” is. As a comparison, the scholastic aptitude test (SAT), a highly popular indicator used for university admissions across the US, shows a mean correlation of about 0.2 with college grade performance^40^ (see Table 1 therein). Also, recently, a comprehensive study of the replicability of research of correlations between personality traits and life outcomes^41^ examined 78 studies in which it was found that the average correlation between these variables was 0.23.

To explore the basic explanatory power of the text variables in our study, we conducted OLS stepwise regressions to predict the four dimensions of EI using all 104 variables. The regression was used to reduce the number of predictors and reduce the risk for overfit. The detailed results of the estimations are given in Appendix S1 and details of the modeling in Appendix S4. We summarize the results in Table 5. The first row in Table 5 lists the adjusted R-squared values of the four regression models in which the dependent variables were the four dimensions of EI. These values ranged between 6 and 10%. This is roughly equivalent to correlations in the range of 0.24–0.32, which in effect is equivalent to the predictive power of the SATs and personality-based behavioral indicators.

**Table 5:** Regression model fit measures. The fit measures in the top row are of OLS stepwise regressions of the whole sample; adjusted R2 measures are given and the F statistic of the models.

	Self-appraisal of emotion	Others’ appraisal of emotion	Use of emotion	Regulation of emotion
Adjusted R^2^ (model F statistic)	9%*** (2.23)	15.4%*** (4.05)	13.2%*** (4.82)	15.6%*** (4.19)

***p < 0.01.

## Discussion and limitations

We have shown that text people generate on an online platform has predictive power regarding their emotional intelligence. We find that EI is positively associated with more incidence of positive emotional expression, but also negatively associated with expressions of negative emotions. The associations with individual text characteristics can be weak, but higher when used together to predict EI. In addition, high EI individuals have some tendency to phrase themselves in a more polite and less confrontational manner than low EI individuals. This is consistent with our finding that high EI individuals also write more about social processes and social behavior than low EI individuals. Using basic statistical models, within the sample, we found that a text-based model explains 9–16% of the variance, which is equivalent to correlations ranging between 0.30 and 0.40. This level of diagnosticity is close to the level of prediction that exists for the SAT, a widely used and influential indicator of academic performance. Also, the level of diagnosticity of the text-based models is similar to the level in which personality indicators predict real-life outcomes, as was tested across 78 behavioral studies^41^. In other words, in our sample, we showed that online public user-generated text may be a useful indicator for EI. In fact, it seems that utilizing a user’s text to infer EI is, in some aspects, equivalent to using a comprehensive and time- and resource-expensive aptitude test. It follows that user-generated text may provide benefit if used as an additional indicator in contexts in which it is important to detect EI, in addition to or instead of extant methods.

Furthermore, we have shown that aspects of the text which are directly related to social behavior and affect predict EI to some extent. This affirms that online texts on digital platforms may be an informative expression of real-life behavior, as this is consistent with the extant psychological literature. In other words, we find that high EI online platform users more prominently discuss topics related to social affairs and emotion. They use more positive emotion when communicating with others, and less negative emotion than low EI individuals. Our findings support the view that online interactions and text can be representative of personality and that concepts which were developed outside the digital realm can be useful when studying digital behavior. An outcome of these findings is that using a tool like the one we present here with online text can enable access to very large and rich data that can allow researchers to study EI in a vast number of individuals, in very fine temporal resolution and in real time. Beyond the potential contribution to theoretical research, our findings open the possibility of building practical applications to help monitor EI in digital settings, such that platforms and community managers can manage online interactions better. Finding individuals with high or low EI, using similar tools, can help platforms set up social interactions more optimally. For example, it could be possible to harness the more-advanced capabilities of certain individuals to mitigate conflict and negative interactions on the one hand, while promoting positive and more productive dynamics, on the other hand.

Another interesting avenue of research could be the use of user-generated text models for EI in therapeutic contexts. For example, it could serve as an important source of information regarding real-life behavior during daily activities. A possible scenario is one in which, conditional on a patient’s consent, a therapist might be able to (carefully) use EI indicators from the patient’s online text. If needed, the indicator can be calculated automatically without exposing the actual text itself to the therapist. This will help improve the accuracy of a useful diagnosis, given that it leverages the patient’s real-life behavior, and could inform the possible direction of therapy^42,43^. Thus, the ability to gain a more comprehensive understanding of a patient’s levels of EI through their texts may be a valuable resource to therapists. It is important to be aware that, in some cases, subjects may be deterred from reporting and reflecting on their real states, and, in some cases, subjects may report on their ought self (as they think they should be), rather than what actually is expressed in their behavior. This phenomenon is even more likely to occur in the context of the patient–therapist relationship where the patient does not have anonymity^44^. Having a more credible measure of EI may be of help in these cases.

A limitation of our findings is the common limitations when using self-report^45^, e.g., social desirability, common method bias, etc. Also, to construct the text variables, we used LIWC. While this is a powerful and widely use text analysis tool, it is limited by the fact that it utilizes word dictionary methods, which is a less flexible approach than, e.g., using large language models. Future studies may study how large language models^46^ (e.g., BERT or GPT) can be used to better extract text properties. Furthermore, given that our focus was on demonstrating that online public texts can predict EI, in this study, we did not control for gender, nationality, or other demographic variables. Future studies should explore the role of demographics in how EI is expressed in online texts.

Notably, the strength of the correlations between text characteristics and EI is also somewhat limited, especially between EI and individual text characteristics. We find statistically significant correlations between text and EI in the range of − 0.20 to 0.20, and that the variance explained ranges between 9 and 16%. Therefore, we propose that these tools be used in conjunction with other tools. We do note that, as described above, the strength of prediction of these tools is in similar magnitude to other widespread indicators (e.g., SATs, personality indicators) used frequently in the industry. Another limitation of our findings is that we only use a certain sample of individuals, on a certain digital platform. This does not clarify whether our results are reproducible on other platforms or for other samples. We hope that future research will test these results on other important platforms and contexts, such that a better generalization can be achieved. Finally, it is important to point out that, while we believe that the ability to detect EI from day-to-day public user activity can be useful to researchers and practitioners, it is vital to consider privacy concerns. The utilization of user-generated text for assessment related to assignment within organizations, or to assist in therapy, should be accompanied by full consent from subjects, after receiving an explanation of the implications for doing so. As is the case for any method which infers information about an individual from their activities, the rights of that individual, including their right to privacy, should be carefully considered^47^.

## Supplementary Information

Supplementary Information.

## Data Availability

The datasets generated and/or analyzed during the current study are available in the figshare repository. The link to the dataset is: https://figshare.com/articles/dataset/emointel_data_to_share_csv/21119914. The link to the readme file: https://figshare.com/articles/online_resource/read_me_emo_inteldata_txt/21119911.
