# Supplementary material for: Characteristics of online user-generated text predict the emotional intelligence of individuals
Source: Sci Rep. 2023 Apr 25;13:6778. doi: 10.1038/s41598-023-33907-4 (PMC10130158; doi:10.1038/s41598-023-33907-4)
Supplement: Supplementary file 1 — Supplementary Information. [file 41598_2023_33907_MOESM1_ESM.docx]

Characteristics of online user-generated text predict emotional intelligence of individuals

Yaniv Dover*^1^, Yair Amichai-Hamburger^2^

. The Hebrew University Business School, Jerusalem, Israel.

2. Reichman University, Herzliya, Israel.

Supplementary materials

**S1. OLS Estimation results for the four dimensions of emotional intelligence**

Below are the regression estimation results for each of the four dimensions of emotional intelligence. In each regression, only variables with significant (p value ≤ 0.05) were used as independent variables.

|  | **self EI** | | |
| --- | --- | --- | --- |
| *Predictors* | *Estimates* | *CI* | *p* |
| (Intercept) | 8.58 | 6.53 – 10.62 | **<0.001** |
| Clout | -0.10 | -0.14 – -0.05 | **<0.001** |
| Dic | 0.08 | -0.01 – 0.17 | 0.084 |
| Linguistic | -0.24 | -0.43 – -0.04 | **0.016** |
| function | 0.20 | 0.02 – 0.38 | **0.032** |
| i | -0.38 | -0.55 – -0.20 | **<0.001** |
| they | -0.42 | -0.80 – -0.04 | **0.032** |
| det | -0.12 | -0.23 – 0.00 | 0.058 |
| auxverb | -0.36 | -0.61 – -0.11 | **0.005** |
| negate | -0.29 | -0.65 – 0.07 | 0.111 |
| verb | 0.38 | 0.16 – 0.61 | **0.001** |
| adj | 0.15 | -0.05 – 0.35 | 0.149 |
| power | -0.49 | -0.91 – -0.07 | **0.023** |
| cogproc | 0.12 | -0.04 – 0.27 | 0.139 |
| insight | -0.46 | -0.72 – -0.20 | **0.001** |
| tentat | -0.21 | -0.42 – -0.01 | **0.037** |
| differ | -0.40 | -0.72 – -0.09 | **0.012** |
| memory | -1.31 | -2.30 – -0.32 | **0.010** |
| Affect | -1.32 | -2.67 – 0.03 | 0.056 |
| tone pos | 1.30 | -0.02 – 2.63 | 0.054 |
| tone neg | 1.47 | 0.10 – 2.85 | **0.036** |
| emotion | 2.70 | 1.02 – 4.39 | **0.002** |
| emo pos | -2.55 | -4.24 – -0.87 | **0.003** |
| emo neg | -3.74 | -5.65 – -1.84 | **<0.001** |
| emo anx | 0.85 | -0.11 – 1.81 | 0.082 |
| emo anger | 1.56 | 0.10 – 3.03 | **0.037** |
| swear | 1.05 | -0.14 – 2.24 | 0.083 |
| Social | 0.48 | 0.21 – 0.76 | **0.001** |
| socbehav | -0.46 | -0.78 – -0.14 | **0.006** |
| female | -0.26 | -0.57 – 0.06 | 0.113 |
| male | -0.31 | -0.62 – -0.01 | **0.041** |
| Culture | -0.65 | -1.38 – 0.08 | 0.082 |
| politic | 0.95 | 0.04 – 1.86 | **0.041** |
| tech | 0.70 | -0.08 – 1.48 | 0.077 |
| Lifestyle | -1.42 | -2.55 – -0.29 | **0.014** |
| leisure | 1.29 | 0.15 – 2.44 | **0.027** |
| home | 1.34 | 0.10 – 2.58 | **0.034** |
| work | 1.48 | 0.39 – 2.57 | **0.008** |
| money | 1.14 | 0.18 – 2.10 | **0.020** |
| relig | 1.53 | 0.32 – 2.75 | **0.014** |
| Physical | 0.41 | 0.13 – 0.69 | **0.005** |
| illness | -1.13 | -2.09 – -0.17 | **0.022** |
| mental | -1.58 | -2.57 – -0.60 | **0.002** |
| sexual | -0.93 | -1.73 – -0.13 | **0.022** |
| food | -0.75 | -1.15 – -0.34 | **<0.001** |
| curiosity | 0.42 | -0.17 – 1.01 | 0.164 |
| attention | -0.35 | -0.85 – 0.15 | 0.170 |
| focusfuture | -0.42 | -0.65 – -0.18 | **<0.001** |
| conj | -0.12 | -0.28 – 0.05 | 0.166 |
| Observations | 606 | | |
| R^2^ / R^2^ adjusted | 0.161 / 0.089 | | |

Table S1: OLS regression of stepwise regression of text variables – dependent variable: Self Appraisal of emotion (SEA)

|  | **others EI** | | |
| --- | --- | --- | --- |
| *Predictors* | *Estimates* | *CI* | *p* |
| (Intercept) | 2.42 | 0.83 – 4.00 | **0.003** |
| Clout | 0.06 | 0.02 – 0.10 | **0.004** |
| Authentic | -0.03 | -0.05 – -0.01 | **0.008** |
| Tone | 0.02 | -0.00 – 0.04 | 0.074 |
| Linguistic | 0.14 | 0.04 – 0.25 | **0.008** |
| pronoun | -0.61 | -0.93 – -0.28 | **<0.001** |
| i | 0.70 | 0.37 – 1.03 | **<0.001** |
| we | 0.57 | 0.08 – 1.06 | **0.022** |
| ipron | 0.56 | 0.25 – 0.87 | **<0.001** |
| article | -0.24 | -0.40 – -0.08 | **0.003** |
| prep | -0.14 | -0.30 – 0.01 | 0.072 |
| adverb | -0.28 | -0.43 – -0.13 | **<0.001** |
| verb | -0.21 | -0.37 – -0.05 | **0.010** |
| adj | -0.27 | -0.43 – -0.10 | **0.001** |
| quantity | -0.21 | -0.37 – -0.04 | **0.016** |
| Drives | -0.40 | -0.63 – -0.17 | **0.001** |
| achieve | 0.59 | 0.17 – 1.00 | **0.006** |
| certitude | 0.50 | 0.19 – 0.82 | **0.002** |
| memory | -0.99 | -1.92 – -0.05 | **0.039** |
| emotion | 1.51 | 0.43 – 2.60 | **0.006** |
| emo pos | -1.45 | -2.59 – -0.31 | **0.013** |
| emo neg | -1.51 | -2.78 – -0.24 | **0.020** |
| emo anx | 0.68 | -0.09 – 1.46 | 0.083 |
| prosocial | 0.39 | 0.02 – 0.76 | **0.040** |
| polite | -0.45 | -0.90 – -0.01 | **0.046** |
| socrefs | 0.24 | -0.01 – 0.50 | 0.063 |
| family | -0.53 | -1.06 – -0.01 | **0.046** |
| female | 0.25 | -0.04 – 0.54 | 0.094 |
| money | 0.23 | 0.04 – 0.41 | **0.018** |
| health | -0.29 | -0.59 – 0.01 | 0.058 |
| want | -0.64 | -1.15 – -0.12 | **0.015** |
| fatigue | 2.99 | 0.10 – 5.89 | **0.042** |
| reward | -1.15 | -1.94 – -0.37 | **0.004** |
| space | 0.29 | 0.12 – 0.46 | **0.001** |
| focuspast | 0.17 | 0.03 – 0.31 | **0.019** |
| focuspresent | 0.15 | -0.02 – 0.32 | 0.085 |
| focusfuture | 0.33 | 0.07 – 0.59 | **0.012** |
| Observations | 606 | | |
| R^2^ / R^2^ adjusted | 0.204 / 0.154 | | |

Table S2: OLS regression of stepwise regression of text variables – dependent variable: Others Appraisal of emotion (OEA)

|  | **use EI** | | |
| --- | --- | --- | --- |
| *Predictors* | *Estimates* | *CI* | *p* |
| (Intercept) | 4.25 | 3.61 – 4.89 | **<0.001** |
| Tone | 0.02 | 0.00 – 0.03 | **0.019** |
| function | -0.06 | -0.10 – -0.03 | **<0.001** |
| shehe | 0.17 | -0.02 – 0.36 | 0.080 |
| ipron | 0.11 | -0.01 – 0.23 | 0.065 |
| Drives | 2.45 | -0.10 – 5.00 | 0.060 |
| affiliation | -2.27 | -4.79 – 0.25 | 0.077 |
| achieve | -2.20 | -4.67 – 0.27 | 0.081 |
| power | -2.37 | -4.88 – 0.13 | 0.063 |
| discrep | 0.20 | 0.01 – 0.40 | **0.045** |
| emo neg | -0.53 | -1.04 – -0.01 | **0.044** |
| emo anx | 1.18 | 0.39 – 1.96 | **0.003** |
| emo sad | -1.52 | -2.80 – -0.25 | **0.019** |
| Culture | 18.20 | -0.83 – 37.22 | 0.061 |
| politic | -17.79 | -36.78 – 1.21 | 0.066 |
| ethnicity | -18.28 | -37.14 – 0.58 | 0.058 |
| tech | -18.23 | -37.25 – 0.79 | 0.060 |
| money | 0.23 | 0.03 – 0.43 | **0.027** |
| death | -0.70 | -1.46 – 0.06 | 0.072 |
| allure | 0.14 | 0.02 – 0.25 | **0.026** |
| Perception | -0.15 | -0.32 – 0.03 | 0.096 |
| attention | 0.61 | 0.12 – 1.11 | **0.014** |
| space | 0.25 | 0.01 – 0.49 | **0.040** |
| memory | -1.04 | -2.00 – -0.08 | **0.034** |
| time | 0.11 | 0.01 – 0.22 | **0.039** |
| Observations | 606 | | |
| R^2^ / R^2^ adjusted | 0.166 / 0.132 | | |

Table S3: OLS regression of stepwise regression of text variables – dependent variable: Use of emotion (UOE)

|  | **reg EI** | | |
| --- | --- | --- | --- |
| *Predictors* | *Estimates* | *CI* | *p* |
| (Intercept) | 4.20 | 1.31 – 7.09 | **0.004** |
| Analytic | 0.02 | -0.00 – 0.04 | 0.083 |
| Clout | -0.04 | -0.09 – -0.00 | **0.042** |
| BigWords | -0.04 | -0.10 – 0.02 | 0.160 |
| ppron | 0.33 | 0.05 – 0.60 | **0.021** |
| i | -0.46 | -0.80 – -0.13 | **0.006** |
| they | -0.53 | -0.86 – -0.20 | **0.002** |
| ipron | 0.14 | 0.03 – 0.26 | **0.017** |
| affiliation | -0.46 | -0.82 – -0.10 | **0.013** |
| Cognition | -1.62 | -3.07 – -0.17 | **0.028** |
| allnone | 1.68 | 0.17 – 3.19 | **0.029** |
| cogproc | 2.11 | 0.59 – 3.62 | **0.007** |
| insight | -0.56 | -0.84 – -0.27 | **<0.001** |
| cause | -0.56 | -0.89 – -0.22 | **0.001** |
| discrep | -0.45 | -0.76 – -0.14 | **0.004** |
| tentat | -0.48 | -0.71 – -0.25 | **<0.001** |
| certitude | -0.50 | -0.85 – -0.16 | **0.004** |
| differ | -0.50 | -0.77 – -0.22 | **<0.001** |
| memory | -1.74 | -2.65 – -0.83 | **<0.001** |
| Affect | -0.16 | -0.35 – 0.03 | 0.095 |
| tone pos | 0.27 | 0.06 – 0.48 | **0.013** |
| emo sad | -1.51 | -2.66 – -0.37 | **0.010** |
| Social | 0.79 | 0.13 – 1.45 | **0.019** |
| socbehav | -0.85 | -1.51 – -0.18 | **0.012** |
| moral | 0.80 | 0.06 – 1.53 | **0.034** |
| socrefs | -0.70 | -1.38 – -0.03 | **0.041** |
| Culture | -1.02 | -1.67 – -0.37 | **0.002** |
| politic | 1.11 | 0.31 – 1.92 | **0.007** |
| tech | 0.94 | 0.26 – 1.61 | **0.006** |
| work | 0.30 | 0.13 – 0.48 | **0.001** |
| illness | -0.55 | -1.29 – 0.20 | 0.149 |
| lack | 1.16 | -0.33 – 2.64 | 0.126 |
| attention | 0.31 | -0.07 – 0.68 | 0.109 |
| space | 0.19 | 0.08 – 0.30 | **0.001** |
| auditory | 0.50 | 0.00 – 0.99 | **0.048** |
| Drives | 0.20 | -0.03 – 0.44 | 0.094 |
| Observations | 606 | | |
| R^2^ / R^2^ adjusted | 0.205 / 0.156 | | |

Table S4: OLS regression of stepwise regression of text variables – dependent variable: Regulation of emotion (ROA)

**S2. List of text variables (generated using LIWC 2022):**

For explanations of the meaning of each text variables, see the documentation of LIWC 2022.

The List of 104 words:

| WC | Analytic | Clout | Authentic | Tone | focuspresent | BigWords | Dic | Linguistic | function | focusfuture |
| --- | --- | --- | --- | --- | --- | --- | --- | --- | --- | --- |
| pronoun | ppron | i | we | you | shehe | they | ipron | det | article | number |
| prep | auxverb | adverb | conj | negate | verb | adj | quantity | Drives | affiliation | achieve |
| power | cognition | allnone | cogproc | insight | cause | discrep | tentat | certitude | differ | memory |
| affect | Tone_pos | Tone_neg | emotion | Emo_pos | Emo_neg | Emo_anx | Emo_anger | Emo_sad | swear | social |
| socbehav | prosocial | polite | conflict | moral | comm | socrefs | family | friend | female | male |
| culture | politic | ethnicity | tech | lifestyle | leisure | home | work | money | relig | physical |
| health | illness | wellness | mental | substances | sexual | food | death | need | want | acquire |
| lack | fulfill | fatigue | reward | risk | curiosity | allure | perception | attention | motion | space |
| visual | auditory | feeling | time | focuspast |  |  |  |  |  |  |

S3. WLEIS scale description

**Measuring emotional intelligence (WLEIS)**

Items in the WLEIS were generated, tested, and cross-validated through a rigorous development process^14^. Subjects were asked to complete a 16-item survey measuring four dimensions of emotional intelligence: (1) Self appraisal (SEA), (2) Others appraisal (OEA), (3) Use of emotion (UOE) and (4) Regulation of emotion (ROE). Each dimension was in fact the mean across its four items. For example, the SEA dimension was calculated as the average of items 1 to 4.

The 16 items in WLEIS are as follows:

Self-Emotion Appraisal (SEA)

1. I have a good sense of why I have certain feelings most of the time.

2. I have a good understanding of my own emotions.

3. I really understand what I feel.

4. I always know whether or not I am happy.

Others Emotion Appraisal (OEA)

5. I always know my friends’ emotions from their behavior.

6. I am a good observer of others’ emotions.

7. I am sensitive to the feelings and emotions of others.

8. I have good understanding of the emotions of people around me.

Use of Emotion (UOE)

9. I always set goals for myself and then try my best to achieve them.

10. I always tell myself I am a competent person.

11. I am a self-motivated person.

12. I always encourage myself to try my best.

Regulation of Emotion (ROE)

13. I am able to control my temper so that I can handle difficulties

rationally.

14. I am quite capable of controlling my own emotions.

15. I can always calm down quickly when I am very angry.

16. I have good control of my own emotions.

S4. Empirical Analysis

For the analysis in the paper, we used Pearson correlations to assess the correlation between each of text variables and each of the four emotional intelligence dimensions. For the analyses presented in the paper, we used basic OLS regressions in the following form:

${EI}_{i}^{j}=B_{0}+\sum_{k} B_{k}\cdot T_{k}+\varepsilon_{i}^{j}.$ (1)

In Eq. (1), ${EI}_{i}^{j}$ denotes one of the four dimensions of emotional intelligence, *j* ($j=1,2,3,4$) for user *i*, $T_{k}$ stands for the 104 text variables ($k=1,2,\ldots,104$), and $\varepsilon_{i}^{j}$ is the error per each emotional intelligence dimension and user. To optimize the model and feature selection, we use stepwise regression.

S5. Table of variables that are not directly under the Affect and Social category.

**Note**: we only included in Table S5 the variables which we find are correlated with EI. For the full description of LIWC’s variable see the original paper. In the table, we denoted the meta-categories (Physical, Perception, etc.).

| **Variable/Dictionary description** | **Variable name** | **Explanation/examples** |
| --- | --- | --- |
| **Summary Variables** |  |  |
| Word count | WC | Total word count |
| Analytical thinking | Analytic | Metric of logical, formal thinking |
| Clout | Clout | Language of leadership, status |
| Authentic | Authentic | Perceived honesty, genuineness |
| Emotional tone | Tone | Degree or positive (negative) tone |
| Big words | BigWords | Percent words 7 letters or longer |
| **Linguistic Dimensions** |  |  |
| Total pronouns | pronoun | I, you, that, it |
| Personal pronouns | ppron | I, you, my, me |
| 1st person singular | i | I, me, my, myself |
| 1st person plural | we | we, our, us, lets |
| 2nd person | you | you, your, u, yourself |
| 3rd person singular | shehe | he, she, her, his |
| 3rd person plural | they | they, their, them, themsel* |
| Determiners | det | the, at, that, my |
| Articles | article | a, an, the, alot |
| Numbers | number | one, two, first, once |
| Prepositions | prep | to, of, in, for |
| Negations | negate | not, no, never, nothing |
| Common adjectives | adj | more, very, other, new |
| Quantities | quantity | all, one, more, some |
| **Psychological Processes** |  |  |
| Drives | Drives | we, our, work, us |
| Affiliation | affiliation | we, our, us, help |
| Achievement | achieve | work, better, best, working |
| Power | power | own, order, allow, power |
| Insight | insight | know, how, think, feel |
| Causation | cause | how, because, make, why |
| Discrepancy | discrep | would, can, want, could |
| Differentiation | differ | but, not, if, or |
| Memory | memory | remember, forget, remind, forgot |
| **Culture** |  |  |
| Culture | Culture | car, united states, govern*, phone |
| Politics | politic | united states, govern*, congress*, |
| Ethnicity | ethnicity | american, french, chinese, indian |
| Technology | tech | car, phone, comput*, email* |
| **Lifestyle** |  |  |
| Lifestyle | lifestyle | work, home, school, working |
| Work | work | work, school, working, class |
| Money | money | business*, pay*, price*, market* |
| **Physical** |  |  |
| Physical | physical | medic*, food*, patients, eye* |
| Health | health | medic*, patients, physician*, health |
| Mental health | mental | mental health, depressed, suicid*, trauma |
| Sexual | sexual | sex, gay, pregnan*, dick |
| Death | death | death*, dead, die, kill |
| **Human states** |  |  |
| Want | want | want, hope, wanted, wish |
| Acquire | acquire | get, got, take, getting |
| Lack | lack | don’t have, didn’t have, *less, hungry |
| Fulfilled | fulfill | enough, full, complete, extra |
| Fatigue | fatigue | tired, bored, don’t care, boring |
| **Human motives** |  |  |
| Reward | Reward | opportunity, win, gain, benefit |
| Curiosity | curiosity | scien*, look* for, research*, wonder |
| Allure | allure | have, like, out, know |
| **Perception** |  |  |
| Perception | Perception | in, out, up, there |
| Attention | attention | look, look* for, watch, check |
| Space | space | in, out, up, there |
| Feeling | feeling | feel, hard, cool, felt |
| **Time orientation** |  |  |
| Future focus | focusfuture | will, going to, have to, may |
